# Impact of a genetic counseling requirement prior to genetic testing

**DOI:** 10.1186/s12913-018-2957-5

**Published:** 2018-03-07

**Authors:** David D. Stenehjem, Trang Au, Amy M. Sainski, Hillevi Bauer, Krystal Brown, Johnathan Lancaster, Vanessa Stevens, Diana I. Brixner

**Affiliations:** 1 Department of Pharmacotherapy, College of Pharmacy, University of Utah, Salt Lake City, UT USA; 2 Huntsman Cancer Institute, Salt Lake City, UT USA; 3 Myriad Genetic Laboratories, Inc, Salt Lake City, UT USA; 4 Department of Internal Medicine, Division of Epidemiology University of Utah, Salt Lake City, UT USA; 5 Personalized Health Care Program, University of Utah, Salt Lake City, UT USA; 6 Department of Pharmacy Practice and Pharmaceutical Sciences, University of Minnesota, College of Pharmacy, 1110 Kirby Drive, 232 Life Science, Duluth, MN 55812 USA

**Keywords:** Genetic testing, Patient survey, Cancer screening, Breast cancer susceptibility genes, Hereditary breast and ovarian cancer syndrome

## Abstract

**Background:**

Genetic counseling by a Genetic Counselor (GC) is a requirement prior to genetic testing for cancer susceptibility genes (GC-mandate policy) for some insurers. This study evaluated the impact of this policy from the patient perspective.

**Methods:**

Surveys were sent to individuals for whom their insurer ordered genetic testing for the cancer susceptibility genes *BCRA1* and *BRCA2* over a 1 year time period that spanned the introduction of a GC-mandate policy. Responses were assessed by time period (before/after policy introduction) and genetic test completion.

**Results:**

The surveys were completed by 1247/4950 (25.7%) eligible individuals. After policy introduction, there was no change in the proportion of respondents who completed genetic testing (*p* = 0.13) or had a mutation (*p* = 0.55). Overall decisional conflict (uncertainty or feeling uninformed) around genetic testing did not change after policy introduction (*p* = 0.16), but was significantly higher among respondents who did not complete genetic testing (*p* < 0.01). Although a larger proportion of respondents saw a GC after policy introduction (*p* < 0.01), fewer did so to better understand their test results (*p* < 0.01). The proportion of respondents who did not see a GC due to insurance issues/requirements and time restraints was higher among those tested after policy introduction or who did not complete genetic testing (*p* < 0.01). In multivariate analysis, respondents with a household income of $25,000 or greater were 3-times more likely to complete testing.

**Conclusions:**

A GC-mandate policy did not improve decisional conflict or increase the number of deleterious mutations identified and low-income respondents were less likely to complete testing. On the contrary, insurance requirements and time constraints may be preventing individuals at risk from receiving appropriate testing.

**Electronic supplementary material:**

The online version of this article (10.1186/s12913-018-2957-5) contains supplementary material, which is available to authorized users.

## Background

Genetic testing for variants in cancer predisposition genes has become common clinical practice for hereditary cancer risk assessment. As such, professional society guidelines incorporate widely agreed upon criteria to identify patients appropriate for testing as well as gene-specific recommendations for risk-reducing medical management [1, 2]. However, there are some concerns that the growing demand for genetic testing may cause an increase in inappropriate testing among patients who do not meet guidelines [3]. This may create additional health care costs with uncertain impact on the short and long term benefit to patients.

In response to the potential rise in inappropriate testing, there is a growing move towards mandatory genetic counseling from a board-certified Genetic Counselor (GC) or Geneticist prior to *BReast CAncer* gene 1 (*BRCA1)* and *BRCA2*) testing [4, 5]. Genetic counseling provides many benefits to patients, including the identification of patients appropriate for testing, patient education, and appropriate medical management [6]. While these services have traditionally been provided by GCs, the growing demand for genetic testing has surpassed the capacity of these specialists [7–9]. The traditional referral model for genetic testing is also known to suffer from poor patient compliance due to poor understanding of insurance coverage [10] as well as concerns about accessibility, logistics and/or cost [11]. Overall, anywhere from 33 to 87% of patients referred to a GC do not follow-through [12–14].

In response to these known barriers, many health care providers are incorporating collaborative models for genetic counseling. These models require additional genetics education for non-specialists and incorporate genetic counseling and testing by medical and gynecologic oncologists, surgeons, and primary care providers [8, 9, 15–17]. Many professional medical societies have also released statements supporting genetic counseling and testing as part of routine care [18–20]. Despite this enhanced genetics training and evidence of increased appropriate testing, some of the largest health care plans in the U.S. have a requirement for genetic counseling by a certified GC prior to genetic testing [21]. Although such policies may encourage access to the knowledge and expertise of these specialists, this may also place an undue burden on patient time and finances as well as delay or prevent risk-reducing medical management. As such, the intended advantages of a mandatory GC requirement must be balanced against the unintended consequence that patients at increased cancer risk may not be tested.

The aim of this study was to evaluate the patient level impact of an insurance-mandated requirement for genetic counseling by a GC or board certified geneticist prior to genetic testing for *BRCA1* and *BRCA2* (GC-mandate policy). This was done by evaluating patient survey responses regarding personal experience with genetic testing and GC consultation, as well as decisional conflict (i.e. feeling uninformed or uncertain about testing decisions). Responses were compared according to time period (before or after GC-mandate policy introduction) and genetic test completion.

## Methods

### Study cohort

This is an analysis of survey responses from patients for whom genetic testing for *BRCA1* and *BRCA2* (*BRCA1/2* testing) was ordered between July 2013 and June 2014 from a single commercial testing laboratory (Myriad Genetic Laboratories, Inc., Salt Lake City, UT). *BRCA1/2* testing included targeted testing for known familial or founder mutations as well as comprehensive sequencing and large-rearrangement analysis for *BRCA1* and *BRCA2*.

Individuals were eligible for inclusion if they had a confirmed order for a *BRCA1/2* test, were 18 years or older at the time of testing, and were covered by one of three health plans that instituted a GC-mandate policy during the time period of this study. Patients were included regardless of whether their *BRCA1/2* test was cancelled (no test result reported). Patients were excluded if they could not be contacted by mail or email, were deceased, did not respond to the survey materials within 3 months, or returned the survey with less than 50% of the questions completed.

### Patient recruitment and survey

Patients were surveyed to explore perceptions of genetic testing before and after the introduction of the GC-mandate policy. IRB approval was obtained from the University of Utah (00084014). A waiver of authorization to receive protected health information (names and contact information) from the testing laboratory was granted along with a waiver of documentation of informed consent to allow participants to provide consent through the act of returning of the survey materials. IRB-approved informed consent, recruitment materials and the survey were emailed to all eligible patients with a valid email address for online completion via REDCap, a University of Utah-supported secure web application. Study materials were mailed to eligible patients who did not complete the survey online or did not have a valid email address.

The survey developed for this study included five main categories: 1) sociodemographic factors and personal/family cancer history, 2) *BRCA1/2* testing process (rationale, ordering health care provider, test completion and results), 3) use of genetic counseling services, and 4) decisional conflict [22] using a 5-point Likert scale adapted from Katapodi et al. 2011. The full survey is provided in the supplemental materials, Additional file 1 .

### Statistical analysis

Survey responses were assessed using chi-squared or Fisher’s exact test, as appropriate, stratified by time period (before and after GC-mandate policy introduction) and by self-reported completion of *BRCA1/2* testing. Decisional conflict questions were scored with point values from 0 to 4 from the five-point Likert responses with higher point values indicating greater decisional conflict. The values from all decisional conflict questions were averaged per patient and multiplied by 25 to yield a mean decisional conflict score for each patient with possible values from 0 to 100. Adjusted mean decisional conflict scores were generated by least squares means method after correction for completion of *BRCA1/2* testing, GC-mandate period, and visit with a GC prior to testing. Logistic regression analyses were performed to assess associations between the completion of *BRCA1/2* testing and sociodemographics, presence of a GC-mandate policy, and pre-test genetic counseling visit.

## Results

### Study recruitment

**Fig. 1:**
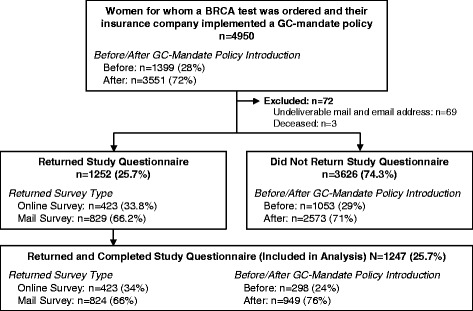
Survey response

There were 4950 eligible individuals for whom genetic testing was ordered during the study time period (Fig. 1). This included 1399 (28%) individuals with *BRCA1/2* tests ordered before and 3551 (72%) individuals with *BRCA1/2* tests ordered after GC-mandate policy introduction. A total of 1247 (25.7%) eligible individuals returned completed surveys. Among all survey responders, 298 (24%) individuals had *BRCA1/2* tests ordered before GC-mandate policy introduction and 949 (76%) individuals had *BRCA1/2* tests ordered thereafter.

### Demographics

Table 1 summarizes the sociodemographic information for all survey responders. There were no statistically significant differences in ethnicity among survey responders tested before or after GC-mandate policy introduction, and the majority (82.4%) of individuals self-reported as Caucasian. There was a significant difference in age among survey responders, with a shift towards younger ages among those whose tests were ordered after policy introduction (*p* = 0.01). There was no difference in household income before and after policy introduction, with 49.9% of survey responders reporting a household income of $100,000 or greater. Personal and family cancer history was not different among survey responders before and after the GC-mandate was introduced.

**Table 1:** Demographic and clinical features and genetic testing rationale

	Total N (%)	GC Requirement Policy			*BRCA1/2* Testing		
		Before N (%)	After N (%)	*P*-value	Tested N (%)	Not Tested N (%)	*P*-value
Total	1247	297 (23.8)	940 (75.4)	n/a	966 (77.5)	271 (21.7)	n/a
Gender (F)	1227 (98.4)	293 (98.3)	934 (98.4)	0.99	950 (98.3)	267 (98.5)	0.29
Ethnicity							
Caucasian	1028 (82.4)	250 (83.9)	778 (82.0)	0.27	801 (82.9)	219 (80.8)	0.26
Hispanic/Latino	83 (6.7)	13 (4.4)	70 (7.4)		58 (6.0)	24 (8.9)	
Black or African American	69 (5.5)	18 (6.0)	51 (5.4)		54 (5.6)	15 (5.5)	
Other	64 (5.1)	17 (5.7)	47 (5.0)		50 (5.2)	13 (4.8)	
Age Range (years)							
18–35	165 (13.2)	26 (8.7)	139 (14.7)	0.01	125 (12.9)	36 (13.3)	0.47
36–45	347 (27.8)	78 (26.2)	269 (28.4)		269 (27.8)	77 (28.4)	
46–55	412 (33.0)	98 (32.9)	314 (33.1)		323 (33.4)	87 (32.1)	
56+	318 (25.5)	94 (31.5)	224 (23.6)		224 (25.6)	68 (25.1)	
Household Income							
< $24,999	29 (2.3)	8 (2.7)	21 (2.2)	0.88	15 (1.6)	14 (5.2)	0.011
$25,000–$49,999	149 (12.0)	33 (11.1)	116 (12.2)		113 (11.7)	35 (12.9)	
$50,000–$99,999	395 (31.7)	100 (33.6)	295 (31.1)		309 (32)	83 (30.6)	
$100,000+	622 (49.9)	146 (49.0)	476 (50.2)		493 (51)	125 (46.1)	
Missing/Declined	52 (4.2)	11 (3.7)	41 (4.3)		36 (3.7)	14 (5.2)	
Personal History							
Any Breast Cancer	347 (27.8)	93 (31.2)	254 (26.8)	0.14	307 (31.8)	38 (14)	< 0.0001
Any Ovarian Cancer	29 (2.3)	5 (1.7)	24 (2.5)	0.40	24 (2.5)	5 (1.8)	0.53
Any Other Cancer	52 (4.2)	14 (4.7)	38 (4.0)	0.60	37 (3.8)	14 (5.2)	0.34
Cancer Free	824 (66.1)	191 (64.1)	633 (66.7)	0.41	601 (62.2)	217 (80.1)	< 0.0001
Family History							
Any Breast Cancer	504 (40.4)	120 (40.3)	384 (4.0)	0.95	383 (39.6)	118 (43.5)	0.25
Ovarian Cancer	97 (7.8)	26 (8.7)	71 (7.5)	0.48	84 (8.7)	12 (4.4)	0.014
Any Other Cancer	222 (17.8)	45 (15.1)	177 (18.7)	0.16	150 (15.5)	71 (26.2)	< 0.0001
Cancer Free	367 (29.4)	92 (30.9)	27 (29.0)	0.53	294 (30.4)	71 (26.2)	0.17
*BRCA1/2* Test Requester							
Any Doctor	891 (71.5)	193 (64.8)	698 (73.6)	0.0034	693 (71.7)	189 (69.7)	0.52
Any Patient	392 (31.4)	120 (40.3)	272 (28.7)	0.0002	309 (32)	81 (29.9)	0.51
Any Neither	17 (1.4)	6 (2.0)	11 (1.2)	0.26	3 (0.3)	14 (5.2)	< 0.0001
Any Other	41 (3.3)	11 (3.7)	30 (3.2)	0.65	34 (3.5)	7 (2.6)	0.43
Top Reasons for Pursuing *BRCA1/2* Testing							
To determine risks for family members	605 (48.5)	146 (49.0)	459 (48.4)	0.85	474 (49.1)	126 (46.5)	0.45
To help plan cancer screening	491 (39.4)	117 (39.3)	374 (39.4)	0.96	367 (38)	118 (43.5)	0.10
To help plan cancer treatment	205 (16.4)	48 (16.1)	157 (16.5)	0.86	190 (19.7)	13 (4.8)	< 0.0001
Because mutations are a common cause of breast cancer	148 (11.9)	27 (9.1)	121 (12.8)	0.09	126 (13)	19 (7.0)	0.004

Categories are non-exclusive

After the GC-mandate policy was introduced, there was a significant increase in the proportion of genetic tests requested by the physician (64.8% before vs. 73.6% after, *p* < 0.01) and a decrease in the proportion requested by the patient (40.3% before vs. 28.7% after, *p* < 0.01). Table 1 also shows that there were no significant changes in the top reasons for *BRCA1/2* testing before and after the policy introduction, with about half of survey responders citing a desire to determine cancer risks for family members (full details in Additional file 2: Table S1).

There were several significant differences in patient demographics when *BRCA1/2* test completion was considered (Table 1). Household incomes were significantly lower among survey responders who did not complete *BRCA1/2* testing (*p* = 0.011). In addition, a higher proportion of survey responders with complete *BRCA1/2* testing had a personal history of breast cancer (31.8% tested vs. 14.0% not tested, *p* < 0.01) and family history of ovarian cancer (8.7% tested vs. 4.4% not tested, *p* = 0.014). Conversely, a lower proportion of survey responders with completed *BRCA1/2* testing had no personal history of cancer (62.2% tested vs. 80.1% not tested, *p* < 0.01) and a family history of a cancer other than breast or ovarian (15.5% tested vs. 26.2% not tested, *p* < 0.01). There were also significant differences in the reason for *BRCA1/2* testing among those with completed tests and those without (Table 1).

### Decisional conflict

Decisional conflict was evaluated by survey questions assessing the extent that respondents felt uncertain or uninformed during the genetic testing process, where a higher mean score corresponds to more decisional conflict (Table 2). There were no significant changes in nearly all of the decisional conflict categories before and after GC-mandate policy introduction, including the proportion of patients who were unsure what to do (*p* = 0.24), needed more advice (*p* = 0.22), or felt they made an informed choice (*p* = 0.47). There was an increase in the proportion of patients who agreed/strongly agreed that they were aware of the choices they had in the decision to get *BRCA1/2* testing after the introduction of the GC-mandate policy (*p* = 0.04) (Table 4). However, the mean score for this question was not statistically different (0.81 before vs. 0.77 after, *p* = 0.50).

**Table 2:** Decisional Conflict

		GC Requirement Policy			*BRCA1/2* Testing		
	Total N (%)	Before N (%)	After N (%)	*P*-value	Tested N (%)	Not Tested N (%)	*P*-value
This decision was hard for me							
Strongly Disagree (0 points)	509 (40.8%)	127 (42.6%)	382 (40.3%)	0.17	439 (47.7%)	70 (31%)	< 0.0001
Disagree (1)	365 (29.3%)	90 (30.2%)	275 (29.0%)		285 (31%)	80 (35.4%)	
Neither Disagree nor Agree (2)	105 (8.4%)	17 (5.7%)	88 (9.3%)		71 (7.7%)	34 (15%)	
Agree (3)	127 (10.2%)	28 (9.4%)	99 (10.4%)		96 (10.4%)	31 (13.7%)	
Strongly Agree (4)	31 (2.5%)	7 (2.4%)	24 (2.5%)		23 (2.5%)	8 (3.5%)	
Do Not Know	9 (0.7%)	5 (1.7%)	4 (0.4%)		6 (0.7%)	3 (1.3%)	
Missing/declined	101 (8.1%)	24 (8.1%)	77 (8.1%)		46 (4.8%)	45 (16.6%)	
Mean Score (SD)	0.93 (1.10)	0.83 (1.05)	0.96 (1.11)	0.11	0.86 (1.08)	1.22 (1.14)	< 0.0001
I was unsure what to do in this decision							
Strongly Disagree (0 points)	479 (38.4%)	119 (39.9%)	360 (37.9%)	0.24	424 (46.2%)	55 (24.7%)	< 0.0001
Disagree (1)	402 (32.2%)	98 (32.9%)	304 (32.0%)		323 (35.2%)	79 (35.4%)	
Neither Disagree nor Agree (2)	113 (9.1%)	20 (6.7%)	93 (9.8%)		75 (8.2%)	38 (17%)	
Agree (3)	105 (8.4%)	27 (9.1%)	78 (8.2%)		66 (7.2%)	39 (17.5%)	
Strongly Agree (4)	25 (2.0%)	2 (0.7%)	23 (2.4%)		19 (2.1%)	6 (2.7%)	
Do Not Know	16 (1.3%)	6 (2.0%)	10 (1.1%)		10 (1.1%)	6 (2.7%)	
Missing/declined	107 (8.6%)	26 (8.7%)	81 (8.5%)		49 (5.1%)	48 (17.7%)	
Mean Score (SD)	0.91 (1.03)	0.81 (0.96)	0.93 (1.05)	0.08	0.80 (0.98)	1.36 (1.12)	< 0.0001
I was aware of the choices I had in this decision							
Strongly Disagree (4 points)	35 (2.8%)	8 (2.7%)	27 (2.9%)	0.04	24 (2.6%)	11 (4.9%)	< 0.0001
Disagree (3)	38 (3.1%)	11 (3.7%)	27 (2.9%)		21 (2.3%)	17 (7.6%)	
Neither Disagree nor Agree (2)	82 (6.6%)	28 (9.4%)	54 (5.7%)		57 (6.2%)	25 (11.1%)	
Agree (1)	485 (38.9%)	111 (37.3%)	374 (39.4%)		368 (40.3%)	117 (52%)	
Strongly Agree (0)	483 (38.7%)	108 (36.2%)	375 (39.5%)		434 (47.5%)	49 (21.8%)	
Do Not Know	15 (1.2%)	8 (2.7%)	7 (0.7%)		9 (1%)	6 (2.7%)	
Missing/declined	109 (8.7%)	24 (8.1%)	85 (9.0%)		53 (5.5%)	46 (17.0%)	
Mean Score (SD)	0.78 (0.91)	0.81 (0.91)	0.77 (0.92)	0.50	0.69 (0.88)	1.16 (0.98)	< 0.0001
I needed more advice and information about the choices							
Strongly Disagree (0 points)	301 (24.1%)	71 (23.8%)	230 (24.2%)	0.22	264 (28.8%)	37 (16.6%)	< 0.0001
Disagree (1)	378 (30.3%)	88 (29.5%)	290 (30.6%)		318 (34.7%)	60 (26.9%)	
Neither Disagree nor Agree (2)	254 (20.4%)	62 (20.8%)	192 (20.2%)		197 (21.5%)	57 (25.6%)	
Agree (3)	129 (10.3%)	31 (10.4%)	98 (10.3%)		87 (9.5%)	42 (18.8%)	
Strongly Agree (4)	57 (4.6%)	11 (3.7%)	46 (4.9%)		39 (4.3%)	18 (8.1%)	
Do Not Know	20 (1.6%)	10 (3.4%)	10 (1.1%)		11 (1.2%)	9 (4%)	
Missing/declined	108 (8.7%)	25 (8.4%)	83 (8.7%)		50 (5.2%)	48 (17.7%)	
Mean Score (SD)	1.31 (1.12)	1.28 (1.10)	1.32 (1.13)	0.64	1.23 (1.10)	1.66 (1.16)	< 0.0001
I feel I made an informed choice							
Strongly Disagree (4 points)	21 (1.7%)	6 (2.0%)	15 (1.6%)	0.47	14 (1.5%)	7 (3.1%)	< 0.0001
Disagree (3)	24 (1.9%)	6 (2.0%)	18 (1.9%)		11 (1.2%)	13 (5.8%)	
Neither Disagree nor Agree (2)	1 (0.1%)	0 (0.0%)	1 (0.1%)		45 (4.9%)	51 (22.7%)	
Agree (1)	386 (31.0%)	93 (31.2%)	293 (30.9%)		290 (31.7%)	96 (42.7%)	
Strongly Agree (0)	596 (47.8%)	135 (45.3%)	461 (48.6%)		550 (60%)	46 (20.4%)	
Do Not Know	18 (1.4%)	8 (2.7%)	10 (1.1%)		6 (0.7%)	12 (5.3%)	
Missing/declined	106 (8.5%)	23 (7.7%)	83 (8.7%)		50 (5.2%)	46 (17.0%)	
Mean Score (SD)	0.64 (0.85)	0.67 (0.88)	0.62 (0.85)	0.42	0.50 (0.78)	1.22 (0.94)	< 0.0001
Decisional Conflict Score^a^, mean (SD)	22.8 (18.2)	22.1 (18.0)	23.0 (18.3)	0.46	20.5 (17.1)	33.0 (19.5)	< 0.0001
Adjusted Decisional Conflict Score, mean (SE)^b^	22.5 (17.3)	22.0 (1.31)	22.6 (0.79)	0.16	20.3 (0.69)	32.7 (1.36)	< 0.0001

^a^Average score across all decisional conflict questions multiplied by 25

^b^Adjusted scores control for GC-mandate period, *BRCA1/2* testing completions, and visit with a genetic counselor prior to testing

When the decisional conflict was evaluated according to *BRCA1/2* test completion, scores were higher across all categories among patients who did not complete *BRCA1/2* testing (*p* < 0.01) (Table 2). The mean decisional conflict score across all five questions was similar before and after the GC-mandate (22.1 vs. 23.0, *p* = 0.46) and was statistically lower in patients who were *BRCA1/2* tested vs not tested (20.5 vs. 33.0, *p* < 0.01).

Adjusted mean decisional conflict scores generated by least squares means method were estimated to assess the relationship between the GC-mandate time period, visit with a GC prior to testing, and completion of genetic testing. This regression analysis confirmed respondents who completed *BRCA1/2* testing had lower overall decisional conflict scores relative to those who did not complete testing (20.3 vs. 32.7, *p* < 0.01), independent of seeing a GC prior to testing and the GC-mandate period (Table 2). No statistical difference was observed before (22.5) or after (22.0) the GC mandate in the adjusted model (*p* = 0.16).

### BRCA1/2 testing

Overall, 966 survey responders reported that they completed *BRCA1/2* testing (Table 3). After GC-mandate policy introduction, there were no significant changes in the proportion of respondents who completed testing (81.5% before vs. 76.2% after, *p* = 0.13) or who were found to carry a pathogenic *BRCA1* or *BRCA2* mutation (10.7% before vs. 8.7% after, *p* = 0.55). There were no significant differences in the most commonly reported uses of genetic test results before and after the policy change (Table 3), where ‘talked with family members about results’ and ‘routine breast cancer screenings’ were the most common uses. Full details are provided in Additional file 2: Table S2.

**Table 3:** Genetic testing completion, results and use of information

		GC Requirement Policy		
	Total N (%)	Before N (%)	After N (%)	*P*-value
*BRCA1/2* Testing Completion	*n* = 1247	*n* = 297	*n* = 940	
Yes	966 (77.5%)	243 (81.5%)	723 (76.2%)	0.13
No	271 (21.7%)	54 (18.1%)	217 (22.9%)	
Unknown/Missing/Declined	10 (0.8%)	1 (0.3%)	9 (1%)	
*BRCA1/2* Test Results	*n* = 966	*n* = 243	*n* = 723	
Mutation	89 (9.2%)	26 (10.7%)	63 (8.7%)	0.55
No Mutation	821 (85.0%)	204 (84.0%)	615 (85.3%)	
Results Uncertain	19 (2.0%)	6 (2.5%)	13 (1.8%)	
Do not Understand Results	8 (0.8%)	0 (0.0%)	8 (1.1%)	
Unknown	8 (0.8%)	2 (0.8%)	6 (0.8%)	
Missing/Declined	21 (2.2%)	5 (2.1%)	16 (2.2%)	
Most commonly reported use of genetic testing results^a^	n = 966	n = 243	n = 723	
Talked with Family Members about Results	590 (47.3%)	147 (60.5%)	443 (61.3%)	0.42
Routine Breast Cancer Screenings	329 (26.4%)	71 (29.2%)	258 (35.7%)	0.25
Plan Cancer Treatment	173 (13.9%)	42 (17.3%)	131 (18.1%)	0.90
Additional Breast Cancer Screening with MRI utilized	53 (4.3%)	10 (4.1%)	43 (6.0%)	0.38
Surgery to Remove Breast or Ovaries to prevent Cancer	40 (3.2%)	11 (4.5%)	29 (4.0%)	0.59
Other	39 (3.1%)	17 (7.0%)	22 (3.0%)	0.003
Most commonly reported reasons for why *BRCA1/2* testing was not completed^a^	*n* = 271	*n* = 54	*n* = 217	
Insurance issues/requirements	165 (60.9%)	39 (72.2%)	126 (58.1%)	0.06
Costs	129 (47.6%)	38 (70.4%)	91 (41.9%)	0.0002
Testing not recommended by GC	56 (20.7%)	3 (5.6%)	53 (24.4%)	0.002
Testing recommended for another family member	24 (8.9%)	4 (7.4%)	20 (9.2%)	0.79
Likelihood low so decided against testing	18 (6.6%)	1 (1.9%)	17 (7.8%)	0.14

Categories are non-exclusive

Among survey responders who did not complete *BRCA1/2* testing, the proportion that cited cost as a contributing factor, decreased after GC-mandate policy introduction (70.4% before vs. 41.9% after, *p* < 0.01) (Table 3). The proportion of respondents who did not complete *BRCA1/2* testing because a GC did not recommend it increased after policy introduction (5.6% before vs. 24.4% after; *p* < 0.01).

### Genetic counseling

The proportion of respondents who received genetic counseling by a GC was higher among those tested after GC-mandate policy introduction (28.9% before vs. 75.5% after, *p* < 0.01) or who completed genetic testing (71.3% tested vs. 39.5% not tested, *p* < 0.01) (Table 4). A higher proportion of patients who saw a GC after the GC-mandate policy was introduced did so due to insurance requirements (16.3% before vs. 80.7% after, *p* < 0.01); however, this change was not observed according to *BRCA1/2* test completion (71.3% tested vs. 79.4% not tested, *p* = 0.16). Conversely, there was a decrease in the proportion of patients for whom *BRCA1/2* tests were ordered after GC-mandate policy introduction who saw a GC to better understand their test results (47.7% before vs. 28.6% after, *p* < 0.01) or because it was recommended by their doctor (64.0% before vs. 22.6% after, *p* < 0.01). Similar trends were observed for respondents who completed *BRCA1/2* testing.

**Table 4:** Genetic Counseling

		GC Requirement Policy			*BRCA1/2* Testing		
	Total N (%)	Before N (%)	After N (%)	P-value	Tested N (%)	Not Tested N (%)	P-value
Received Genetic Counseling							
Total	n = 1247	n = 297	n = 940		n = 966	n = 271	
Yes	802 (64.3%)	86 (28.9%)	716 (75.5%)	< 0.0001	689 (71.3%)	107 (39.5%)	< 0.0001
No	389 (31.2%)	193 (64.8%)	196 (20.7%)		243 (25.2%)	144 (53.1%)	
Unknown	40 (3.2%)	12 (4.0%)	28 (3.0%)		27 (2.8%)	12 (4.4%)	
Most Commonly Reported Reasons for Receiving Genetic Counseling^a^							
Total	*n* = 802	*n* = 86	*n* = 716		*n* = 689	*n* = 107	
Insurance Requirement	592 (73.8%)	14 (16.3%)	578 (80.7%)	< 0.0001	504 (73.1%)	85 (79.4%)	0.16
Chance to better Understand the Test Results	246 (30.7%)	41 (47.7%)	205 (28.6%)	0.0003	224 (32.5%)	21 (19.6%)	0.0054
Recommended by Doctor	217 (27.1%)	55 (64.0%)	162 (22.6%)	< 0.0001	196 (28.4%)	21 (19.6%)	0.049
Most Commonly Reported Reasons for Not Receiving Genetic Counseling^a^							
Total	*n* = 389	*n* = 193	*n* = 196		n = 243	*n* = 144	
Was not Aware GC was Available	175 (45.1%)	122 (63.2%)	53 (27.2%)	< 0.0001	145 (59.9%)	28 (19.4%)	< 0.0001
Insurance Issues/Requirements	88 (22.6%)	22 (11.4%)	66 (33.7%)	< 0.0001	17 (7%)	70 (48.6%)	< 0.0001
Inconvenience of Additional Step	30 (7.7%)	10 (5.2%)	20 (10.3%)	0.06	12 (5%)	18 (12.5%)	0.0087
Time Restraints	29 (7.5%)	5 (2.3%)	24 (12.3%)	0.0003	12 (5%)	17 (11.8%)	0.016
Not Interested in Pursuing	24 (6.2%)	14 (7.3%)	10 (5.1%)	0.38	16 (6.6%)	8 (5.6%)	0.68
Cost of Appointment	23 (5.9%)	7 (3.6%)	16 (8.2%)	0.06	4 (1.7%)	19 (13.2%)	< 0.0001
Did you receive a benefit from genetic counseling?							
Total	*n* = 632	*n* = 103	*n* = 529		*n* = 521	*n* = 110	
Yes	503 (79.6%)	80 (77.7%)	423 (80.0%)	0.60	438 (84.1%)	64 (58.2%)	< 0.0001
No	129 (20.4%)	23 (22.3%)	106 (20.0%)		83 (15.9%)	46 (41.8%)	
Would you recommend genetic counseling to family or friends?							
Total	*n* = 633	*n* = 102	*n* = 531		*n* = 524	*n* = 109	
Yes	574 (90.7%)	99 (97.6%)	475 (89.5%)	0.014	479 (91.4%)	95 (87.2%)	0.18
No	59 (9.3%)	3 (2.9%)	56 (10.6%)		45 (8.6%)	14 (12.8%)	
Would you utilize genetic counseling in the future?							
Total	*n* = 626	n = 103	*n* = 523		*n* = 518	*n* = 108	
Yes	548 (87.5%)	95 (92.2%)	453 (86.6%)	0.10	454 (87.6%)	94 (87.0%)	0.86
No	78 (12.5%)	8 (7.8%)	70 (13.4%)		64 (12.4%)	14 (13%)	

Categories are non-exclusive

Among survey responders who did not see a GC prior to genetic testing, the proportion that was not aware that a GC was available decreased after policy introduction (63.2% before vs. 27.2% after, *p* < 0.01). This was accompanied by an increase in the proportion of patients who did not see a GC due to insurance issues/requirements (11.4% before vs. 33.7% after, *p* < 0.01) or time restraints (2.3% before vs. 12.3% after, *p* < 0.01) (Table 4  and full details provided in Additional file 2: Table S3). The same trends were observed by test completion, where the proportion of respondents who did not see a GC due to insurance issues (7.0% tested vs. 48.6% not tested, *p* < 0.0001) or time restraints (5.0% tested vs. 11.8% not tested, *p* = 0.016) was higher among those who did not complete *BRCA1/2* testing. In addition, the proportion of respondents who did not see a GC due to the inconvenience of an added step increased, but did not reach statistical significance, among those tested after policy introduction (5.2% before vs. 10.3% after, *p* = 0.06) or who did not complete *BRCA1/2* testing (5.0% tested vs. 12.5% not tested, *p* < 0.01).

### Logistic regression for completion of genetic testing

In multivariate analysis (Additional file 2: Table S4), individuals were more likely to complete genetic testing before the GC-mandate policy was introduced (OR 3.7, 95% CI 2.42 = 5.75). In addition, household income was a significant predictor of completion of genetic testing, where survey responders with a household income of $25,000 or greater were 3.08–3.60 times more likely to complete testing relative to responders with a household income of less than $25,000. Consultation with a GC prior to testing was significantly predictive of genetic test completion (OR 6.35, 95% CI 4.47–9.10, *p* < 0.01). In addition, several clinical features were predictive of genetic test completion including personal history of breast cancer (OR 2.46, 95% CI 1.65–3.76, *p* < 0.01) and family history of ovarian cancer (OR 1.99, 95% CI 1.05–4.11, *p* < 0.0335). Patients with a personal or family history of a cancer other than breast or ovarian were less likely to complete genetic testing (OR 0.49, 95% CI 0.34–0.72, *p* < 0.01).

## Discussion

With the increased clinical use of genetic testing for hereditary cancer risk, there are concerns that inappropriate testing may rise. In response, there has been a shift by some insurance companies to require counseling by a GC prior to genetic testing. However, a previous study by Whitworth et al. showed that introduction of a GC-mandate policy decreased *BRCA1/2* testing among patients with increased cancer risks [23]. Here we evaluated the patient impact of a GC-mandate policy by analyzing survey responses from individuals for whom *BRCA1/2* testing was ordered before and after policy introduction.

Overall, the data presented here show minimal evidence of utility for the GC-mandate policy as demonstrated by decisional conflict scores and the deleterious mutation rate. After the introduction of a GC-mandate policy, there was little to no change in the decisional conflict, or extent to which respondents felt uncertain or uninformed during the genetic testing process. Survey responders who were tested after policy introduction were more aware of the choices they had in the decision to get genetic testing; however, there was no change in the proportion of patients who found the decision to get genetic testing difficult to make, were unsure what to do, needed more advice, or importantly felt they made an informed choice. It is noteworthy that the majority of survey respondents were Caucasian and almost half reported annual household incomes of at least $100,000. As such, the data presented here may indicate that consultation with a GC may have affirmed patients’ prior knowledge in an educated group of individuals of high socioeconomic class who were likely informed about genetic testing prior to meeting with a GC. While this would not be expected to affect decisional conflict, genetic counseling in this scenario may help solidify patient decisions.

Our study also demonstrates completion of genetic testing influences decisional conflict. Decisional conflict was significantly higher in all categories assessed among patients who did not complete genetic testing relative to those who were tested. Even when the overall decisional conflict scores were adjusted to account for seeing a GC, scores were significantly higher for those who did not complete *BRCA1/2* testing. This suggests that responders who completed *BRCA1/2* testing had comparatively lower decisional conflict scores because they received a test result and were informed by the results. Conversely, responders without *BRCA1/2* testing may have been more conflicted about testing, since genetic testing was originally ordered and presumably desired by the patient and/or physician but subsequently canceled, provoking feelings of uncertainty regarding the process and their genetic risk for hereditary breast and ovarian cancer since they did not receive a result.

Survey responders indicated insurance, cost, and ‘testing not recommended by a GC’ as the three most common reasons for not completing genetic testing. This is consistent with another prospective study by Hayden et al. that evaluated value of GC prior to genetic testing [24]. In our study, there was a significant increase in responders citing ‘testing not recommended by GC’ after the GC-mandate. While it is unclear whether this is due to the increased proportion of patients who saw a GC, it may support the added value and role of GCs in patient education and selection to aid in testing decisions. A significant decrease in respondents citing costs as a reason for not completing testing was observed after the GC-mandate, which was potentially commensurate to the increase in testing not being recommend by a GC, mitigating the need and cost of testing.

After the introduction of the GC-mandate policy, there was an increase in the proportion of patients who saw a GC, as expected. However, there was no change in the patient-reported deleterious mutation rate. The introduction of a GC-mandate as a pre-approval process for insurers would be expected to reduce inappropriate *BRCA1/2* testing, since one of the roles of the GC is to differentiate between patients for whom genetic testing is or is not appropriate. Improved patient selection for *BRCA1/2* testing would be expected to produce a relative increase in the proportion of deleterious *BRCA1/2* mutations identified in those tested. The absence of change in the deleterious mutation rate suggests that the GC-mandate did not improve patient selection for testing. While this study was not designed to formally assess appropriateness of testing, this finding is consistent with a previous report by Whitworth et al. showing that the laboratory-reported deleterious mutation rate did not change after the GC-mandate policy was introduced [23]. In addition, our multivariate analysis showed that patients were over three times more likely to be tested before policy introduction. Collectively, this suggests that the GC-mandate policy maybe preventing appropriate patients (i.e. those with increased hereditary cancer risk) from being tested. This highlights the importance of carefully evaluating policies and their effect on mitigating inappropriate testing while optimizing selection of patients for appropriate testing.

The benefits of genetic counseling are well described in the literature, and the patient experience with genetic counseling in our study was favorable and similar before and after the GC-mandate. Approximately 80% of respondents in both time periods felt they received benefit from genetic counseling and 87.5% of respondents would utilize genetic counseling in the future. However, there was a decrease in the proportion of patients who would recommend consultation with GC to a family member after the GC-mandate. Also, the proportion of patients who saw a GC to better understand their test results or who were recommended to see a GC by their physician decreased after policy introduction, likely due to the mandate for genetic counseling and education prior to testing. Among survey responders who did not see a GC, there was an increase after policy introduction in the proportion that cited insurance issues/requirements, cost, time constraints, and inconvenience as reasons for not receiving genetic counseling. Although GC consultation by phone was available as part of the GC-mandate policy, the concerns cited here are consistent with previously reported limitations and barriers of the traditional referral model for genetic testing [10, 11].

Several factors were found to be predictive of whether genetic testing would be completed, including consultation with a GC, as well as personal history of breast cancer and family history of ovarian cancer. Income was positively predictive, where survey responders who reported a household income of $25,000 or greater were more than three times as likely to complete genetic testing relative to responders with a household income less than $25,000. This suggests that the GC-mandate policy introduces barriers to care among low-income individuals, which is consistent with preliminary findings by Whitworth et al. that underserved populations may be disproportionately impacted by policy introduction [23].

A few limitations to this analysis are important to describe. Overall, the survey response rate was relatively low at 25.7%. Average survey response rates for studies utilizing data collected from individuals has been reported to be 52.7% [25]. Baruch et al. indicate primary reasons for non-response include failure to deliver survey to target population and individual reluctance to respond [25]. Both reasons may help explain the response rate observed. Between time of genetic testing and survey dissemination, survey participants may have relocated or no longer use the email address provided. More importantly, given the controversy surrounding privacy issues related to genetic testing, individuals may have felt hesitant to respond to our survey originating outside of their health care system and focused on a sensitive area. Although this survey was nationally distributed, study results should nevertheless be interpreted in context of survey responders’ demographics (minimal ethnic diversity, higher reported income).

## Conclusions

Collectively, the data presented here suggest that there is minimal added utility in an insurance mandate requiring consultation with a GC prior to genetic testing. While there was an increase in the proportion of patients who saw a GC, the GC-mandate did not improve decisional conflict, or increase the deleterious mutation rate, and may have contributed to health disparities. As such, alternative models of patient selection for genetic testing should be considered.

## Additional files

Additional file 1: **Table S1.** Demographic and Clinical Features and BRCA1/2 testing rationale, **Table S2.** Reasons why BRCA1/2 testing was not completed, **Table S3.** Genetic Counseling Responses. **Table S4.** Provides the output of logistic regression for odds of completion of BRCA testing based on several variables. (DOCX 46 kb)

Additional file 2: **Study survey.** The study survey instrument disseminated to study participants. (PDF 337 kb)

## Abbreviation

GC: Genetic Counselor
