# Supplementary material for: Impact of a genetic counseling requirement prior to genetic testing
Source: BMC Health Serv Res. 2018 Mar 7;18:165. doi: 10.1186/s12913-018-2957-5 (PMC5842549; doi:10.1186/s12913-018-2957-5)
Supplement: Supplementary file 1 — Table S1. Demographic and Clinical Features and BRCA1/2 testing rationale, Table S2. Reasons why BRCA1/2 testing was not completed, Table S3. Genetic Counseling Responses. Table S4. Provides the output of logistic regression for odds of completion of BRCA testing based on several variables. (DOCX 46 kb) [file 12913_2018_2957_MOESM1_ESM.docx]

# Supplemental Table. 1. Demographic and Clinical Features and *BRCA1/2* testing rationale

|  | **Total**  **N (%)** | **GC Requirement Policy** | | | ***BRCA1/2* Testing** | | |
| --- | --- | --- | --- | --- | --- | --- | --- |
|  |  | **Before**  **N (%)** | **After**  **N (%)** | **P-value** | **Tested**  **N (%)** | **Not Tested**  **N (%)** | **P-value** |
| **Total** | 1247 | 297 (23.8) | 940 (75.4) | n/a | 966 (77.5) | 271 (21.7) | n/a |
| **Gender (F)** | 1227 (98.4) | 293 (98.3) | 934 (98.4) | 0.99 | 950 (98.3) | 267 (98.5) | 0.29 |
| **Current Health** |  |  |  |  |  |  |  |
| Excellent | 321 (25.7) | 66 (22.2) | 255 (26.9) | 0.44 | 248 (25.7) | 71 (26.2) | 0.072 |
| Very Good | 566 (45.4) | 146 (50.0) | 420 (44.3) |  | 452 (46.8) | 110 (40.6) |  |
| Good | 332 (26.6) | 80 (26.9) | 252 (26.6) |  | 249 (25.8) | 80 (29.5) |  |
| Poor | 26 (2.1) | 6 (2.0) | 20 (2.1) |  | 16 (1.7) | 10 (3.7) |  |
| Missing/Declined | 2 (0.2) | 0 (0.0) | 2 (0.2) |  | 1 (0.1) | 0 (0.0) |  |
| **Reasons for Pursuing *BRCA1/2* Testing** |  |  |  |  |  |  |  |
| To determine risks for family members | 605 (48.5) | 146 (49.0) | 459 (48.4) | 0.85 | 474 (49.1) | 126 (46.5) | 0.45 |
| To help plan cancer screening | 491 (39.4) | 117 (39.3) | 374 (39.4) | 0.96 | 367 (38) | 118 (43.5) | 0.10 |
| To help plan cancer treatment | 205 (16.4) | 48 (16.1) | 157 (16.5) | 0.86 | 190 (19.7) | 13 (4.8) | **<0.0001** |
| Because mutations are a common cause of breast cancer | 148 (11.9) | 27 (9.1) | 121 (12.8) | 0.09 | 126 (13) | 19 (7.0) | **0.004** |
| Curiosity | 40 (3.2) | 5 (1.7) | 35 (3.7) | 0.09 | 25 (2.6) | 15 (5.5) | **0.023** |
| Because all women should be tested | 21 (1.7) | 5 (1.7) | 16 (1.7) | 0.99 | 12 (1.2) | 9 (3.3) | **0.031** |
| Do not recall reason | 17 (1.4) | 4 (1.3) | 13 (1.4) | 1.0 | 10 (1) | 7 (2.6) | 0.073 |
| No reason was given | 1 (0.1) | 1 (0.3) | 0 (0.0) | 0.24 | 0 (0) | 1 (0.4) | 0.22 |
| Missing/Declined | 255 (20.5) | 75 (25.2) | 180 (19.0) | **0.02** | 203 (21) | 51 (18.8) | 0.43 |
| **DNA Sample Given (Y)** | 1130 (90.6) | 261 (87.9) | 869 (91.6) | 0.23 | 922 (95.4) | 198 (73.1) | **<0.0001** |

*Categories are non-exclusive.

# Supplemental Table 2. Reasons why *BRCA1/2* testing was not completed*

|  |  | **GC Requirement Policy** | | |
| --- | --- | --- | --- | --- |
|  | **Total**  **N (%)** | **Before**  **N (%)** | **After**  **N (%)** | **P-value** |
| **Total** | 271 | 54 | 217 |  |
| Insurance issues/requirements | 165 (60.9%) | 39 (72.2%) | 126 (58.1%) | 0.06 |
| Costs | 129 (47.6%) | 38 (70.4%) | 91 (41.9%) | **0.0002** |
| Testing not recommended by GC | 56 (20.7%) | 3 (5.6%) | 53 (24.4%) | **0.002** |
| Testing recommended for another family member | 24 (8.9%) | 4 (7.4%) | 20 (9.2%) | 0.79 |
| Likelihood low so decided against testing | 18 (6.6%) | 1 (1.9%) | 17 (7.8%) | 0.14 |
| Fear of health discrimination | 12 (4.4%) | 2 (3.7%) | 10 (4.6%) | 1.0 |
| Different genetic test recommended | 9 (3.3%) | 2 (3.7%) | 7 (3.2%) | 1.0 |
| Concerns about emotional consequences | 6 (2.2%) | 1 (1.9%) | 5 (2.3%) | 1.0 |
| Confused why test needed | 2 (0.7%) | 0 (0.0%) | 2 (0.9%) | 1.0 |
| Discouraged by family member | 1 (0.4%) | 1 (1.9%) | 0 (0.0%) | 0.20 |
| Did not want to know results | 1 (0.4%) | 0 (0.0%) | 1 (0.5%) | 1.0 |
| Other | 40 (14.8) | 5 (9.3%) | 35 (16.1%) | 0.20 |

*Categories are non-exclusive.

# Supplemental Table 3. Genetic Counseling

|  |  | **GC Requirement Policy** | | | ***BRCA1/2* Testing** | | |
| --- | --- | --- | --- | --- | --- | --- | --- |
|  | **Total**  **N (%)** | **Before**  **N (%)** | **After**  **N (%)** | **P-value** | **Tested**  **N (%)** | **Not Tested**  **N (%)** | **P-value** |
| **Received Genetic Counseling** | | | | | | | |
| Total | 1247 | 297 | 940 |  | 966 | 271 |  |
| Yes | 802 (64.3%) | 86 (28.9%) | 716 (75.5%) | **<0.0001** | 689 (71.3%) | 107 (39.5%) | **<0.0001** |
| No | 389 (31.2%) | 193 (64.8%) | 196 (20.7%) |  | 243 (25.2%) | 144 (53.1%) |  |
| Unknown | 40 (3.2%) | 12 (4.0%) | 28 (3.0%) |  | 27 (2.8%) | 12 (4.4%) |  |
| **Reasons for Receiving Genetic Counseling *** | | | | | | | |
| Total | 802 | 86 | 716 |  | 689 | 107 |  |
| Insurance Requirement | 592 (73.8%) | 14 (16.3%) | 578 (80.7%) | **<0.0001** | 504 (73.1%) | 85 (79.4%) | 0.16 |
| Chance to better Understand the Test Results | 246 (30.7%) | 41 (47.7%) | 205 (28.6%) | **0.0003** | 224 (32.5%) | 21 (19.6%) | **0.0054** |
| Recommended by Doctor | 217 (27.1%) | 55 (64.0%) | 162 (22.6%) | **<0.0001** | 196 (28.4%) | 21 (19.6%) | **0.049** |
| Benefit to Family’s Future | 48 (6.0%) | 8 (9.3%) | 40 (5.6%) | 0.17 | 42 (6.1%) | 6 (5.6%) | 0.84 |
| Recommended by Family Member | 22 (2.7%) | 8 (9.3%) | 14 (2.0%) | **0.0012** | 19 (2.8%) | 3 (2.8%) | 1.0 |
| Other | 28 (3.5%) | 12 (14.0%) | 16 (2.2%) | **<0.0001** | 23 (3.3%) | 5 (4.7%) | 0.50 |
| **Reasons for Not Receiving Genetic Counseling** | | | | | | | |
| Total | 389 | 193 | 196 |  | 243 | 144 |  |
| Was not Aware GC was Available | 175 (45.1%) | 122 (63.2%) | 53 (27.2%) | **<0.0001** | 145 (59.9%) | 28 (19.4%) | **<0.0001** |
| Insurance Issues/Requirements | 88 (22.6%) | 22 (11.4%) | 66 (33.7%) | **<0.0001** | 17 (7%) | 70 (48.6%) | **<0.0001** |
| Inconvenience of Additional Step | 30 (7.7%) | 10 (5.2%) | 20 (10.3%) | 0.06 | 12 (5%) | 18 (12.5%) | **0.0087** |
| Time Restraints | 29 (7.5%) | 5 (2.3%) | 24 (12.3%) | **0.0003** | 12 (5%) | 17 (11.8%) | **0.016** |
| Not Interested in Pursuing | 24 (6.2%) | 14 (7.3%) | 10 (5.1%) | 0.38 | 16 (6.6%) | 8 (5.6%) | 0.68 |
| Cost of Appointment | 23 (5.9%) | 7 (3.6%) | 16 (8.2%) | 0.06 | 4 (1.7%) | 19 (13.2%) | **<0.0001** |
| Not Important Enough | 11 (2.8%) | 3 (1.6%) | 8 (4.1%) | 0.13 | 6 (2.5%) | 5 (3.5%) | 0.57 |
| Logistic Concerns | 6 (1.6%) | 2 (1.0%) | 4 (2.1%) | 0.69 | 1 (0.4%) | 5 (3.5%) | **0.029** |
| Anxiety/Emotions Regarding Meeting | 6 (1.6%) | 0 (0.0%) | 6 (3.1%) | **0.03** | 1 (0.4%) | 5 (3.5%) | **0.029** |
| Discouraged by Family Members | 1 (0.3%) | 0 (0.0%) | 1 (0.5%) | N/A | 0 (0%) | 1 (0.7%) | 0.37 |
| Other | 67 (17.3%) | 28 (14.5%) | 39 (20%) | 0.15 | 40 (16.5%) | 27 (18.8%) | 0.58 |
| **Feel Like Made an Informed Choice Regarding *BRCA1/2* Testing*** | | | | | | | |
| Total | 1237 | 297 | 940 |  | 966 | 271 |  |
| Strongly Disagree | 44 (3.6%) | 12 (4%) | 32 (3.4%) | 0.94 | 27 (2.8%) | 17 (6.3%) | **<0.0001** |
| Disagree | 33 (2.7%) | 6 (2%) | 27 (2.9%) |  | 8 (0.8%) | 25 (9.2%) |  |
| Neither Disagree nor Agree | 100 (8.1%) | 28 (9.4%) | 72 (7.7%) |  | 34 (3.5%) | 66 (24.4%) |  |
| Agree | 360 (29.1%) | 85 (28.6%) | 275 (29.3%) |  | 268 (27.7%) | 92 (33.9%) |  |
| Strongly Agree | 656 (53%) | 156 (52.5%) | 500 (53.2%) |  | 612 (63.4%) | 44 (16.2%) |  |
| Do Not Know | 25 (2%) | 4 (1.3%) | 21 (2.2%) |  | 7 (0.7%) | 18 (6.6%) |  |
| Missing/Declined | 19 (1.5%) | 6 (2%) | 13 (1.4%) |  | 10 (1.0%) | 9 (3.3%) |  |
| **If genetic counseling was not a requirement, would you still like to know your test results?** | | | | | | | |
| Total | 228 | 52 | 176 |  |  |  |  |
| Yes | 201 (88.2%) | 47 (90.4%) | 154 (87.5%) | 0.84 | — | — | — |
| No | 6 (2.6%) | 1 (1.9%) | 5 (2.8%) |  | — | — |  |
| Undecided | 21 (9.2%) | 4 (7.7%) | 17 (9.7%) |  | — | — |  |
| **Would you like to receive assistance in finding genetic counseling resources so you could try pursuing genetic testing again?** | | | | | | | |
| Total | 263 | 51 | 212 |  |  |  |  |
| Yes | 113 (43.0%) | 26 (51%) | 87 (41%) | 0.20 | — | — | — |
| No | 150 (57.0% | 25 (49%) | 125 (59%) |  | — | — |  |

*Categories are non-exclusive.

# Supplemental Table 4. Logistic regression for completion of *BRCA1/2* testing

|  | **Odds ratio for completion of BRCA testing** | **95% CI** | **P-Value** |
| --- | --- | --- | --- |
| **Visit with a genetic counselor prior to testing (Y vs N)** | 6.35 | 4.47-9.10 | **<0.0001** |
| **GC-mandate Time Period (Before vs After)** | 3.70 | 2.42-5.75 | **<0.0001** |
| **Personal history of breast cancer (Y vs N)** | 2.46 | 1.65-3.76 | **<0.0001** |
| **Family history of cancer other than breast or ovarian** | 0.49 | 0.34-0.72 | **0.0003** |
| **Family history of ovarian cancer** | 1.99 | 1.05-4.11 | **0.0335** |
| **Income** |  |  |  |
| Less than $24,999 | ref |  |  |
| $25,000-$49,999 | 3.08 | 1.14-8.31 | **0.0262** |
| $50,000-$99,999 | 3.60 | 1.42-9.06 | **0.0072** |
| $100,000+ | 3.37 | 1.35-8.35 | **0.0097** |
