# Supplementary material for: Impact of a genetic counseling requirement prior to genetic testing
Source: BMC Health Serv Res. 2018 Mar 7;18:165. doi: 10.1186/s12913-018-2957-5 (PMC5842549; doi:10.1186/s12913-018-2957-5)
Supplement: Supplementary file 2 — Study survey. The study survey instrument disseminated to study participants. (PDF 337 kb) [file 12913_2018_2957_MOESM2_ESM.pdf]

Before We Get Started

We value your response EVEN IF you did NOT receive BRCA testing

Directions:

Thank you for your help with this survey. Please select **one answer choice** for each question unless the question indicates **Mark all that apply**.

As a token of appreciation, you will be entered into a drawing for an iPad mini once you return this survey **BY MARCH 1, 2016**. In addition, if you are one of the first 1,000 survey participants to return this survey, you will be mailed or emailed a $15 gift card from below. You will be unable to change your selection after you return this survey. These gifts are only available with the return of this mailed survey.

Please pick ONE:

- ☐ $15 Amazon Gift Card
- ☐ $15 Target Gift Card
- ☐ $15 Barnes and Noble Gift Card

Address or email address you wish your gift card sent to:

Email: ____

Address: ______________________

____

____

Definitions:

BRCA Test Definition: Genetic test to see if you have any mutations in your BRCA1 or BRCA2 genes

Genetic Counselor Definition: A licensed professional other than your doctor that discusses your genetic history and risks of possessing mutations.

Process Timeline:

Pre-genetic counseling requirement: BRCA Test Ordered → Sample Taken → BRCA Test Run → BRCA Test Results Delivered

Post-genetic counseling requirement: BRCA Test Ordered → Sample Taken → Genetic Counselor → BRCA Test Run → BRCA Test Results Delivered

About You

1. Gender
   - Male
   - Female
2. Ethnicity
   - Caucasian
   - Hispanic/Latino
   - Black or African American
   - Native American
   - Asian/Pacific Islander
   - Other
3. Age Range
   - 18-25 years old
   - 26-35 years old
   - 36-45 years old
   - 46-55 years old
   - 56-65 years old
   - 66+ years old
4. What category best describes your annual household income?
   - Less than $24,999
   - $25,000 to $49,999
   - $50,000 to $99,999
   - $100,00 or more
5. How would you rate your current health?
   - Excellent
   - Very Good
   - Good
   - Poor
6. At the time you or your physician were considering BRCA testing, were you diagnosed with breast, ovarian, both, or other cancer?
   - Breast
   - Ovarian
   - Both breast and ovarian
   - Other cancer
   - Cancer free (no cancer)
7. At the time of testing, were any of your family members diagnosed with breast, ovarian, both, or other cancer?
   - Breast
   - Ovarian
   - Both breast and ovarian
   - Other cancer
   - Cancer free (no cancer)

BRCA Testing Process and Prior Genetic Counseling Questions

8. Did you or your doctor request BRCA genetic testing?
   - o Myself
   - o Doctor
   - o Neither
   - o Other____
9. If recommended by your doctor or healthcare provider, what was the reason you were told that this test was being recommended? (**Mark all that apply**)
   - ☐ To help plan cancer treatment
   - ☐ To help plan cancer screening
   - ☐ To determine risks for family members
   - ☐ Because mutations are a common cause of breast cancer
   - ☐ Curiosity
   - ☐ Because all women should be tested
   - ☐ No reason was given
   - ☐ I don't recall
10. Did you give a sample (blood or mucosal wash) to be tested for a BRCA mutation?
    - o Yes
    - o No
    - o Unknown
11. At the time you or your doctor requested testing did your health plan require genetic counseling before BRCA testing?
    - o Yes

      If Yes, how did you find out about the genetic counseling requirement?
      - o Doctor
      - o Health Insurance Plan
      - o Genetic Testing Laboratory
      - o Unknown
      - o Other____
    - o No
    - o Unknown
12. Did you receive genetic counseling from a licensed provider (not your doctor) prior to BRCA testing? (more answer choices on next page)
    - o Yes

      If Yes, why did you receive genetic counseling? (**Mark all that apply**)
      - ☐ Insurance requirement
      - ☐ Benefit to family's future
      - ☐ Recommended by doctor or health care provider
      - ☐ Recommended by family member
      - ☐ Chance to better understand the test results or appropriateness of testing
      - ☐ Other____

If Yes, I understand the circumstances that brought me to genetic counseling.

- ○ Do not agree
- ○ Somewhat agree
- ○ Completely agree
- ○ Do not know

○ No

If No, why did you **NOT** receive genetic counseling? (**Mark all that apply**)

- ☐ Time restraints
- ☐ Logistic Concerns (Transportation, Daycare)
- ☐ Anxiety/emotions regarding meeting
- ☐ Insurance issues/requirements
- ☐ Cost of appointment
- ☐ Discouraged by family member
- ☐ Was not aware genetic counseling was available or an option
- ☐ Inconvenience of an additional step
- ☐ Not interested in pursing
- ☐ Not important enough
- ☐ Other____

○ Unknown

BRCA testing results

13. Did you complete BRCA genetic testing?

- ○ Yes – Go to Page 5
- ○ No – Go to Page 6

If you completed BRCA genetic testing

(Please complete if you answered YES to Question 13, go to page 6 if you answered NO to Question 13)

14. Do you recall receiving your BRCA genetic testing results?
    - o Yes
    - o No
15. What were your BRCA test results?
    - o A mutation was found
    - o No mutation was found
    - o The result was uncertain
    - o I do not understand my results
    - o Unknown
16. Did you receive genetic counseling after testing to discuss your BRCA test results?
    - o Yes
    - o No
17. Who all discussed the BRCA test results with you? (**Mark all that apply**)
    - ☐ Doctor
    - ☐ Nurse
    - ☐ Genetic counselor
    - ☐ Other Health Care Provider (Nurse practitioner, Physician’s Asst., etc., Medical Assistant)
    - ☐ Other
    - ☐ Don’t recall
18. Have you used the information from your genetic testing in any of the following ways? (**Mark all that apply**)
    - ☐ The test result helped me plan my cancer treatment
    - ☐ Based on my test result, I had surgery to remove my breasts or ovaries to prevent cancer from developing
    - ☐ Based on my test result, it is appropriate for me to have routine breast cancer screening (annual mammogram and clinical breast exam for women age 40 and over)
    - ☐ Based on my test result, my risk for breast cancer is increased and additional breast cancer screening with breast MRI is appropriate
    - ☐ I have talked to my family members about my test result
    - ☐ Other ways you have used the information: ______________________________
19. Do you feel like you made an informed choice about BRCA testing? (**Choose one**)

☐ Strongly Disagree — ☐ Disagree — ☐ Neither Disagree nor Agree — ☐ Agree — ☐ Strongly Agree

OR

Do Not Know

Refuse to Answer

If you did not complete BRCA genetic testing
(Please complete if you answered NO to Question 13, skip if you answered YES to Question 13)

20. Why did you **NOT** undergo BRCA testing (**Mark all that apply**)
    - ☐ Costs
    - ☐ Testing not recommended by Genetic Counselor
    - ☐ Testing recommended for another family member
      Did that family member get tested? **Circle your answer**: **Yes** **No**
    - ☐ A different genetic test was recommended
      Did you receive those testing results? **Circle your answer**: **Yes** **No**
    - ☐ Concerns about the emotional consequences of the BRCA results
    - ☐ I learned that the likelihood of having a BRCA mutation in my family was low, and I decided not to have testing
    - ☐ Insurance Issues/Requirements
    - ☐ Fear of Health/Life Insurance Discrimination
    - ☐ Discouraged by family member
    - ☐ Did not want to know the results
    - ☐ Confused as to why I needed the test and/or didn't understand implications of the test
    - ☐ Other____

21. If genetic counseling was not a requirement, would you still like to know your test results?
    - o Yes
    - o No
    - o Undecided

22. Do you feel like you made an informed choice about BRCA testing? (**Choose one**)

☐ Strongly Disagree ☐ Disagree ☐ Neither Disagree nor Agree ☐ Agree ☐ Strongly Agree **OR** Do Not Know / Refuse to Answer

23. Do you need more advice and information about your choices for BRCA testing? (**Choose one**)

☐ Strongly Disagree ☐ Disagree ☐ Neither Disagree nor Agree ☐ Agree ☐ Strongly Agree **OR** Do Not Know / Refuse to Answer

24. Would you like to receive assistance in finding genetic counseling resources so you could try pursuing genetic testing again
    - o Yes
    - o No

25. If yes, what are some reasons that you are interested in this information? (**Mark all that apply**)
    - ☐ To help plan cancer treatment
    - ☐ To help plan cancer screening
    - ☐ To determine risks for family members
    - ☐ Because mutations are a common cause of breast cancer
    - ☐ Curiosity
    - ☐ Other____

Complete if you ever saw a genetic counselor at any time

26. Did you receive a benefit from genetic counseling?
    - Yes
    - No
27. Would you recommend genetic counseling to family or friends?
    - Yes
    - No
28. Would you utilize genetic counseling in the future?
    - Yes
    - No

Optional Decisional Conflict Scale

Please indicate how strongly you agree or disagree with these statements about genetic testing.

DC1 This decision was hard for me to make. (Choose one)

☐ Strongly Disagree ☐ Disagree ☐ Neither Disagree nor Agree ☐ Agree ☐ Strongly Agree **OR** Do Not Know / Refuse to Answer

DC2 I was unsure what to do in this decision. (Choose one)

☐ Strongly Disagree ☐ Disagree ☐ Neither Disagree nor Agree ☐ Agree ☐ Strongly Agree **OR** Do Not Know / Refuse to Answer

DC3 I was aware of the choices I had in this decision. (Choose one)

☐ Strongly Disagree ☐ Disagree ☐ Neither Disagree nor Agree ☐ Agree ☐ Strongly Agree **OR** Do Not Know / Refuse to Answer

DC4 I needed more advice and information about the choices. (Choose one)

☐ Strongly Disagree ☐ Disagree ☐ Neither Disagree nor Agree ☐ Agree ☐ Strongly Agree **OR** Do Not Know / Refuse to Answer

DC5 I feel I made an informed choice. (Choose one)

☐ Strongly Disagree ☐ Disagree ☐ Neither Disagree nor Agree ☐ Agree ☐ Strongly Agree **OR** Do Not Know / Refuse to Answer
